# Transcriptomic and proteomic strategies to reveal the mechanism of *Gymnocypris przewalskii* scale development

**DOI:** 10.1186/s12864-024-10047-1

**Published:** 2024-02-03

**Authors:** Baoke Xu, Yanrong Cui, Linlin A., Haichen Zhang, Qinghua Ma, Fulei Wei, Jian Liang

**Affiliations:** 1 State Key Laboratory of Plateau Ecology and Agriculture, Qinghai University, 251 Ningda Road, Xining, 810016 People’s Republic of China; 2 School of Ecological and Environmental Engineering, Qinghai University, 251 Ningda Road, Xining, 810016 People’s Republic of China

**Keywords:** *Gymnocypris przewalskii*, Scale development, Transcriptome, Proteome

## Abstract

**Background:**

Fish scales are typical products of biomineralization and play an important role in the adaptation of fish to their environment. The *Gymnocypris przewalskii* scales are highly specialized, with scales embedded in only specific parts of the dermis, such as the areas around the anal fin and branchiostegite, making *G. przewalskii* an ideal material for biomineralization research. In this study, we aimed to unveil genes and pathways controlling scale formation through an integrated analysis of both transcriptome and proteome, of which *G. przewalskii* tissues of the dorsal skin (no scales) and the rump side skin (with scales) were sequenced. The sequencing results were further combined with cellular experiments to clarify the relationship between genes and signaling pathways.

**Results:**

The results indicated the following: (1) a total of 4,904 differentially expressed genes were screened out, including 3,294 upregulated genes and 1,610 downregulated genes (with a filtering threshold of |log2Fold-Change|> 1 and *p*-adjust < 0.05). The identified differentially expressed genes contained family members such as *FGF*, *EDAR*, *Wnt10*, and *bmp*. (2) A total of 535 differentially expressed proteins (DEPs) were filtered out from the proteome, with 204 DEPs downregulated and 331 DEPs upregulated (with a filtering threshold of |Fold-Change|> 1.5 and *p* < 0.05). (3) Integrated analyses of transcriptome and proteome revealed that *emefp1*, *col1a1*, *col6a2*, *col16a1*, *krt8*, and *krt18* were important genes contributing to scale development and that PI3K-AKT was the most important signaling pathway involved. (4) With the use of the constructed *G. przewalskii* fibroblast cell line, *emefp1*, *col1a1*, *col6a2*, *col16a1*, *krt8*, and *krt18* were confirmed to be positively regulated by the PI3K-AKT signaling pathway.

**Conclusion:**

This study provides experimental evidence for PI3K-AKT controlled scale development in *G. przewalskii* and would benefit further study on stress adaptation, scale biomineralization, and the development of skin appendages.

**Supplementary Information:**

The online version contains supplementary material available at 10.1186/s12864-024-10047-1.

## Introduction

Skin appendages are structures that developed from the ectoderm and the underlying mesenchyme, and usually contain skin glands, bony and keratinous structures [1]. They have a very important role in the survival of vertebrates [2]. Fish scales are typical skin appendages that have a hard bony structure and are located on the surface of the fish body, a derivative unique to fish skin and belonging to the deformed part of the dermis. They have important biological roles such as maintaining the special body shape of fish, reducing external damage from water flow, and preventing pathogenic attacks. The morphology and number of scales are often used as taxonomic features and are also important aspects for identifying age and analyzing growth and development status [3]. There are three types of scales in fish [4]: shield scales in cartilaginous fish (sharks and rays), hard scales in hard-scaled fish (sturgeon, bowfin, finfish, and multifin), and bone scales in most hard-scaled fish. Bone scales are bony structures that evolved from the dermis and are rounded, with the anterior end inserted into the scale capsule and the posterior end exposed outside the skin in a free state, arranged in an interlocking tile pattern. Bone scales are divided into cycloid and ctenoid scales according to the shape of the free posterior margin. Cycloid scales have a smooth and rounded posterior margin and are commonly found in carp-like and herring-like fishes. The posterior edge of the ctenoid scale has serrated protrusions, which are mostly found in fish such as Perciformes. Whether it is a cycloid scale or a ctenoid scale, there are concentric rings on the surface, which can be used to infer the age and growth rate of the fish.

Generally, fish scales can be divided into two layers: the osseous layer is mainly composed of hydroxyapatite and distributed with a small amount of collagen fibers, and the fibrous layer is mainly formed by the intersection of adjacent collagen fibers. However, it was found that the onset location of scale development, the number of scales, and the process of coverage vary considerably among different fishes. For most fish, the body surface is covered with scales, except for the head; for example, carp and grass carp are covered with scales all over their bodies. Only for a few fish do the scales grow on specific parts of the body. The onset location of scale initiation is known to be divided into three categories in different fishes: (1) the onset of scale development is located at the lateral line behind the gill cover, e.g., *Ctenopharyngodon idella*, *Cyprinus carpio*, *Schizothorax davidi*, and *Schizothorax prenanti* [5, 6]. (2) Scales begin at the position of the middle lateral line of the tail, e.g., *Danio rerio* and *Oreochromis mossambicus* [7, 8]. (3) There is another category with an irregular starting point; e.g., in *Mugil soiuy*, scales first appear on the line between the caudal peduncle and the middle of the carapace, and in *Oplegnathus fasciatus* [9], scales appear on the base of the pectoral fin to the posterior edge of the gill cover. The mechanism controlling scale onset is still unclear, and some studies have speculated that it is influenced by epigenetic factors [10, 11], and the pattern of scale initiation may be determined by fish adaptation to their survival environment or by specific genetic regulatory mechanisms.

Fish scale development is a typical biomineralization process that elaborately regulated by complex gene networks. As fish scales, mammalian hair and avian feathers are all skin appendages, even though they are morphologically different, they are part of the skin structure and have the same origin, and they may share similar molecular mechanisms [12]. On the basis of this starting point, scientists have undertaken some useful explorations using different biological materials through evolutionary correlations and accumulated much knowledge on morphogenesis and cytology, such as applying pathways or genes that have an impact on the skin development process to the study of fish scales [13]. For instance, the ectodysplasin A (EDA)/EDA receptor (EDAR) pathway was first discovered in mammals, and defects in this pathway can cause a lack of skin appendages, i.e., sweat gland dysgenesis, in humans and mice [14]; this pathway has also been found in zebrafish and medaka, and mutations in this pathway result in abnormal scale morphology in fish, as evidenced by a partial or complete lack of scale development [15–17]. Studies at the time of carp scale genesis found that both EDA and EDAR genes are specifically expressed in the skin matrix where scales are born but are weakly expressed or not expressed in non-scale regions, and their expression disappears after all scales have developed and formed [18]. The bone morphogenetic protein (BMP) signaling pathway was found to be involved in fin development in zebrafish [19], and our study found that the BMP signaling pathway is indeed involved in the formation of shell biomineralization in *Pinctada maxima* and can regulate the expression of matrix proteins such as KRMP [20]. Defects in the fibroblast growth factor/fibroblast growth factor receptor (FGF/FGFR) signaling pathway can lead to severe genetic disorders, and FGFR mutations in zebrafish lead to a significant reduction in scales. The SHH/PTC signaling pathway is involved in the formation of feathers, morphogenesis and differentiation of the scale tegument, and the formation of cortical fins [21]. However, in general, little is known about the molecular mechanisms underlying their occurrence and subsequent development up to morphogenesis. What is certain is that the formation of fish scale covers is regulated by multiple genes and is a complex regulatory process.

*Gymnocypris przewalskii* grows in an alpine, low-oxygen, and strong UV environment and is a primitive fish in the highland environment [22]. It is a cold-water, brackish endemic fish that evolved under the long-term geographical isolation of Qinghai Lake. This migratory fish plays a key role in the “fish-bird symbiosis” ecosystem of Qinghai Lake [23]. Its spawning period is from May to July each year, and damage to its species resources will seriously affect the balance of the whole ecosystem of the Qinghai Lake. The biodiversity value of *G. przewalskii* is high, and the conservation and effective use of its germplasm resources are necessary. In the process of adapting to the low oxygen, low temperature, and special water environment of the Qinghai-Tibetan Plateau, the scales on *G. przewalskii*’s body surface appear to be specialized, with only a few scales growing behind the gills and on the rump side. This study used transcriptomics and proteomics to sequence and analyze dorsal scaleless skin tissues and anal scaly skin, to screen important genes and signaling pathways that control scale formation in *G. przewalskii*. The relationship between signaling pathways and important genes was further verified using myofibroblast cell lines. This lays a theoretical foundation for exploring the unique adaptation mechanisms of *G. przewalskii* to the plateau environment, as well as its germplasm conservation, and also provides reference data for studying other skin appendages.

## Results

### Scale coverage pattern of *Gymnocypris przewalskii*

**Fig. 1:**
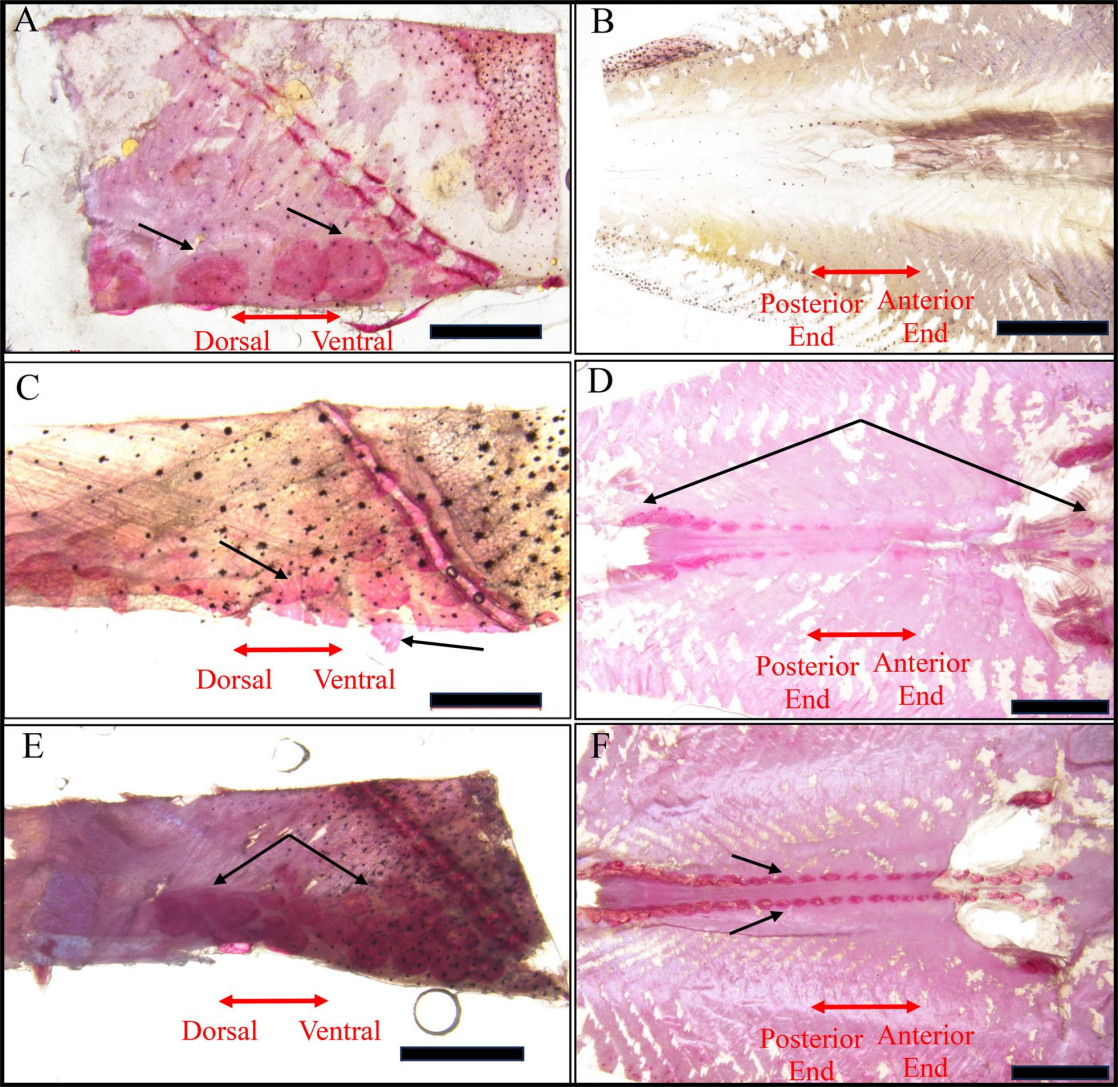
Observation on the scale development in *Gymnocypris przewalskii* juvenile. **A** Observation of scale development at skin area adjacent to the posterior edge of the gill at 70 days post-hatch. **B** Observation of scale development at skin area around the anal fin in juvenile fish at 70 days post-hatch. **C** Observation of scale development at skin area adjacent to the posterior edge of the gill in juvenile fish at 74 days post-hatch. **D** Observation of scale development at skin area around the anal fin in juvenile fish at 74 days post-hatch. **E** Observation of scale development at skin area adjacent to the posterior edge of the gill in juvenile fish at 92 days post-hatch. **F** Observation of scale development at skin area around the anal fin in juvenile fish at 92 days post-hatch. Black arrows indicated scales stained with Alizarin Red S in juvenile fish. Scale bar, 500 μm

Development of scales was observed in *G. przewalskii* juveniles at 70, 74 and 92 days post-hatch in a chromogenic manner, using Alizarin Red S as the staining reagent. It was found that initiation of scales was first observed in juveniles of 70-days-post-hatch at skin area adjacent to the posterior edge of the gill cover (Fig. 1A), when the fish were 52.8 ± 7.2 mm in length. No scale growth was observed in the rest parts of the skin, including tissues around the anal fin (Fig. 1B). Four days later, development of scales was began to observe near the anal fin, loosely aligned along the ventral median line (Fig. 1D). At this time, scales developed behind the grill cover were irregular in arrangement, though they were more densely arranged (Fig. 1C). When the 92-days-post-hatch juveniles were subjected to observation, scales behind the gill cover were found to be aligned orderly (Fig. 1E). These scales were more densely arranged and regular in shape than those grew near the anal fin (Fig. 1F). According to our observation, after the emergence of the posterior gill scales, the scales of the anal and ventral fins appeared almost simultaneously and then grew from the anal fin to the ventral fin.

### Observations on the multistage structure of *Gymnocypris przewalskii* scales

**Fig. 2:**
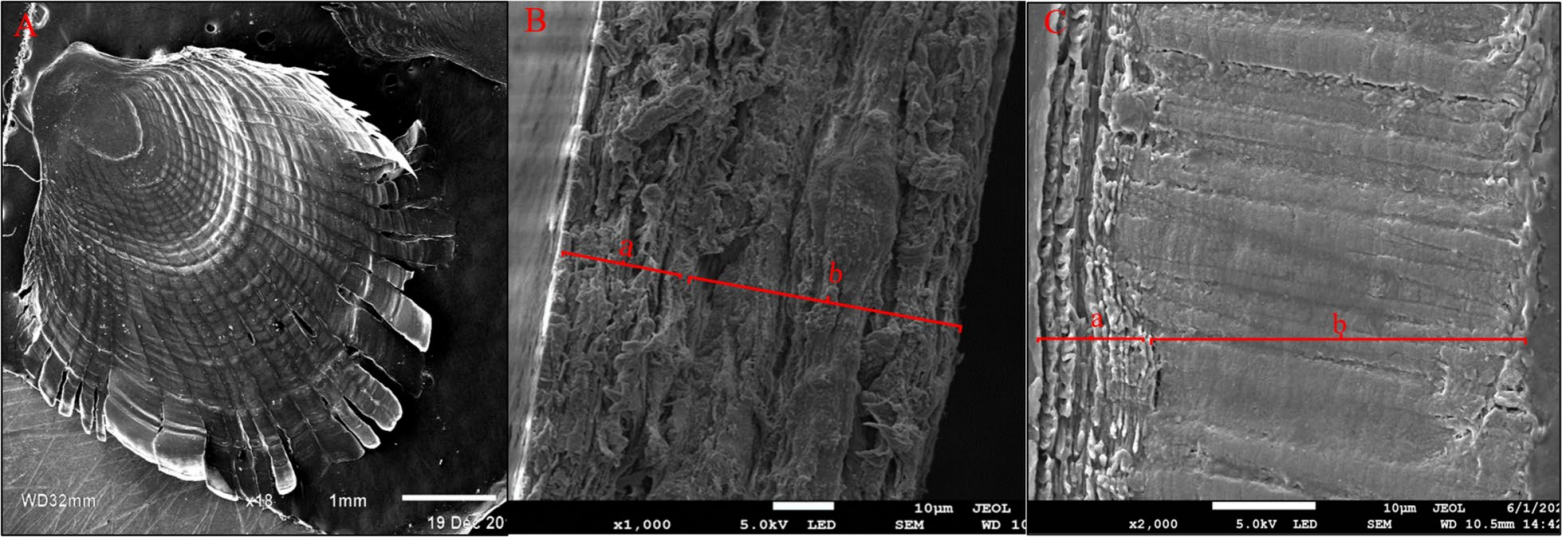
Scanning electron microscopy observation of the scale sections of *Gymnocypris przewalskii*. **A** shows the overall planar structure of scales from *G. przewalskii*; magnification, × 10; scale bar, 1 mm. **B** shows the cross-sectional structure of scales from *G. przewalskii*; magnification, × 1,000; scale bar, 10 μm. **C** shows the longitudinal structure of scales from *G. przewalskii*; magnification, × 2,000; scale bar, 10 μm. a: osteoid layer; b: collagen fiber layer

The cross-sectional scanning electron microscopy results of *G. przewalskii* scales are presented in Fig. 2. *G. przewalskii* scales are cycloid scales (Fig. 2A), which have two layers: the outer layer is the osteoid layer, and the inner layer is the collagen fiber layer. The bone layer is a continuous structure, partially embedded in the collagen fiber layer, which facilitates coordinated deformation between the bone and collagen layers, and the collagen fiber layer has a thin plywood structure and is discontinuous (Fig. 2B, C). The ratio of the thickness of the bony layer to the thickness of the collagen fiber layer at the location of the *G. przewalskii* scale sections was approximately 1:3.5, which indicates that the thickness of the collagen fiber layer at all scale sections was greater than the thickness of the bony layer, which explains the relatively soft *G. przewalskii* scales.

### Transcriptomic sequencing results

Transcriptome sequencing was performed in the skin tissues of S (dorsal area without scales) and M (area around the anal fin with scales). The raw sequence data reported in this paper have been deposited in the Genome Sequence Archive in National Genomics Data Center, China National Center for Bioinformation/Beijing Institute of Genomics, Chinese Academy of Sciences, which are publicly accessible at https://ngdc.cncb.ac.cn/gsa (accession number: CRA010997). After quality control and data filtering, 148,731,780 bp and 149,952,060 bp high-quality clean reads were acquired for S and M. The GC content was 46.61–49.51%, the percentage of Q20 bases was > 97.03%, and the percentage of Q30 bases was > 92.80% (Table 1). 432,316 transcripts and 161,375 unigenes were generated using Trinity software. The mean length of transcripts in the de novo transcriptome assembly was 1,096 bp with an N50 of 1,671 bp. Furthermore, the mean length of unigenes was 934 bp with a N50 of 1,303 bp (Table 2).

**Table 1:** Quality of the sequencing data for transcriptome analysis of scaled (M) and non-scaled skin tissues (S)

Sample	Clean reads	Clean bases	Error (%)	GC content (%)	Q20 (%)	Q30 (%)
M1	41,951,280	6.29G	0.01	48.76	97.35	93.47
M2	49,002,862	7.35G	0.01	47.76	97.4	93.66
M3	58,997,918	8.85G	0.01	49.51	97.5	93.81
S1	50,448,542	7.57G	0.02	46.61	97.03	92.8
S2	51,267,054	7.69G	0.02	46.7	97.08	92.89
S3	47,016,184	7.05G	0.01	47.14	97.76	94.32

**Table 2:** Annotation of unigenes in different databases

Type	Number of unigenes	Percentage (%)
Annotated in Nr	528,20	32.73
Annotated in Nt	148,544	92.04
Annotated in KEGG	21,252	13.16
Annotated in Swiss-Prot	34,976	21.67
Annotated in Pfam	40,574	25.14
Annotated in GO	40,574	25.14
Annotated in KOG	14,143	8.76
Total unigenes	161,375	100

Unigenes with an expression level of FPKM > 0.3 were included in annotation. According to the statistics of nt annotation of the S group, 82,809 (S1), 82,869 (S2), and 79,255 (S3) unigenes were assembled in sum respectively, and only 3817, 3830, 3477 unigenes could not be annotated. As to samples of the M group, 75,065 (M1), 65,937 (M2), and 83,418 (M3) unigenes were assembled in total respectively, and 3057 (M1), 2757 (M2), and 3624 (M3) unigenes were found to contain no annotation information. Then, 32.73% unigenes were matched to proteins in the NR database, followed by 92.04% in the NT database, 13.16% in the KO database, 21.67% in the SwissProt database, 25.14% in the PFAM database, 25.14% in the GO database, and 8.76% in the KOG database.

### Differential gene expression analysis

**Fig. 3:**
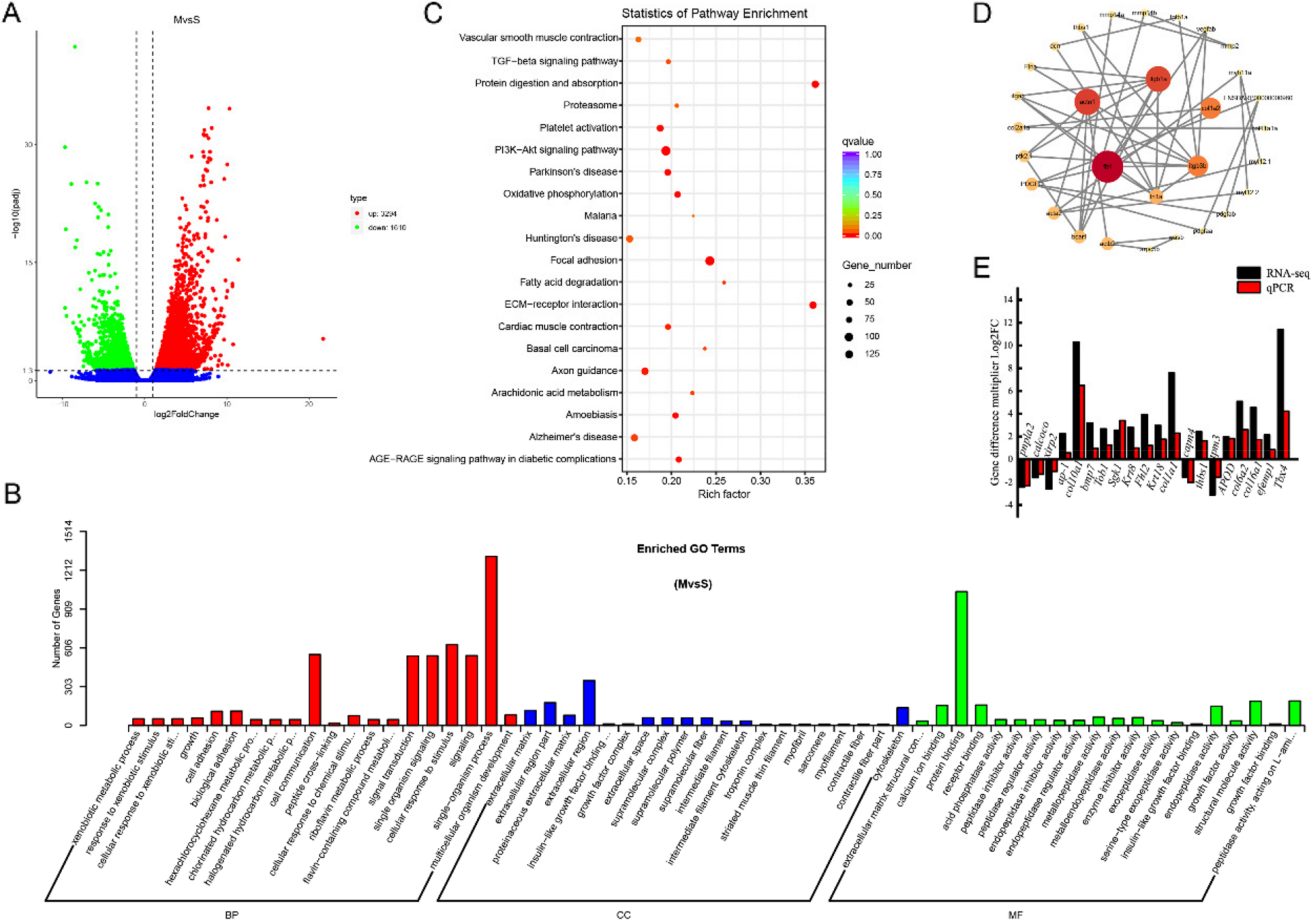
Overview of skin transcriptome data analysis. **A** Differential gene expression in the skin of groups M and S. **B** Gene Ontology classification of differentially expressed genes between groups M and S. **C** KEGG enrichment diagram of differentially expressed genes between groups M and S. **D** DEG protein interaction network. DEG, differentially expressed gene. **E** Comparision of gene expression levels quantified by RNAseq and qPCR in different skin tissues of *G. przewalskii*

Expression analysis revealed that a total of 4,904 differentially expressed genes (DEGs) were identified between the skin around the anal fin (M) and the dorsal skin (S) of *G. przewalskii*, with a filtering threshold of |log2Fold-Change|> 1 and *p*-adjust < 0.05. The number of DEGs up-regulated and down-regulated were 3,294 and 1,610, respectively (Fig. 3A).

### GO enrichment and KEGG signaling pathway analyses

After annotation in the seven databases, GO enrichment analysis was performed for the DEGs identified. The 4904 DEGs were enriched in 88 GO terms, which could be classified into three major categories, i.e. biological process (BP), cellular component (CC), and molecular function (MF). “single organism processes”, “extracellular regions” and “protein binding” were terms that most highly enriched in BP, CC and MF, respectively (Fig. 3B). In the CC category, extracellular matrix, extracellular region part, proteinaceous extracellular matrix, extracellular region, extracellular matrix structural constituent, calcium ion binding, protein binding, suggesting their involvement in scale genesis. The 1610 down-regulated DEGs were mainly enriched in GO terms, such as metabolic process, catalytic activity, organic substance metabolic process, single-organism metabolic process, etc. ( Figure S1, Table S1); The 3294 up-regulated DEGs were mainly enriched in binding, single-organism process, protein binding, biological regulation, and regulation of biological process, etc. (Figure S2; Table S2).

We also performed KEGG enrichment analyses of all the DEGs. The DEGs were annotated to 281 KEGG pathways and significantly enriched in 33 signaling pathways (Table S3). As illustrated in Fig. 3C, protein digestion and absorption was the most significantly enriched pathway, followed by ECM-receptor interaction, focal adhesion, and PI3K-Akt signaling pathway. Ranking by the q-value, the other 17 pathways, i.e. oxidative phosphorylation, AGE-RAGE signaling pathway in diabetic complications, amoebiasis, parkinson’s disease, platelet activation, axon guidance, cardiac muscle contraction, fatty acid degradation, Alzheimer’s disease, arachidonic acid metabolism, basal cell carcinoma, TGF-beta signaling pathway, Huntington’s disease, proteasome, malaria, vascular smooth muscle contraction, were also included in the top-20 most enriched pathways.

Further analysis revealed that down-regulated DEGs were significantly enriched in Oxidative phosphorylation, Parkinson’s disease, Alzheimer’s disease, and Huntington’s disease (Figure S3, Table S4); up-regulated DEGs were significantly enriched in ECM-receptor interaction, Protein digestion and absorption, Focal adhesion, and PI3K-Akt signaling pathway (Figure S4, Table S5).

### Hub genetic analysis

The differential gene interaction network was constructed using String 11.0 (https://version-11-0.string-db.org/), and the top 30 protein–protein interaction network core genes were selected for graphing on the basis of the protein–protein interaction network centrality values (Fig. 3D), with higher values indicating the more critical role of the node in the network. The five highest nodes were *flt4*, *itgβ1a*, *actn1*, *itgβ3b*, and *col1a2*, indicating that these genes may be pivotal genes for scale formation in *G. przewalskii*. Meanwhile, *col2a1*, *mmp2*, *Flna*, *fn1a*, *dcn*, *acta2*, and *pdgfc* may play important roles in scale formation. In addition, the differential genes included FGF gene family members *fgf1*, *fgfr1*, *fgf2*, and *fgfr2*; BMP gene family members *bmp4*, *bmp7*, and *bmpr2*; Wnt pathway-related genes *wnt1*, *wnt4*, *wnt5*, and *wnt10*; and EDA pathway-related genes *eda*, *edar*, and *eda2r*.

### Validation of RNA-Seq through qRT-PCR

To verify the accuracy of the transcriptomic results, we used real-time fluorescence quantitative PCR to analyze the relative gene expression of 20 selected genes between the skin tissues around the anal fin (with scale) and those of the dorsal area (without scale), and we plotted the results as presented in Fig. 3E. The expression of 20 randomly selected genes in both tissues was consistent with the trend of transcriptomic sequencing results. Therefore, transcriptomic data analysis is reliable.

### Differential expression analysis of proteins

To further investigate the molecular mechanism of scale formation in *G. przewalskii*, skin from two sites identical to transcriptome analysis was selected for proteomic sequencing. The skin around the anal fin (with scales) (group D, three replicates: D1, D2, and D3), and the skin on the back (without scales) (group C, three replicates: C1, C2, and C3) were subjected to comparative proteomic analysis, and differential proteins and signaling pathways were identified between the two groups.

**Fig. 4:**
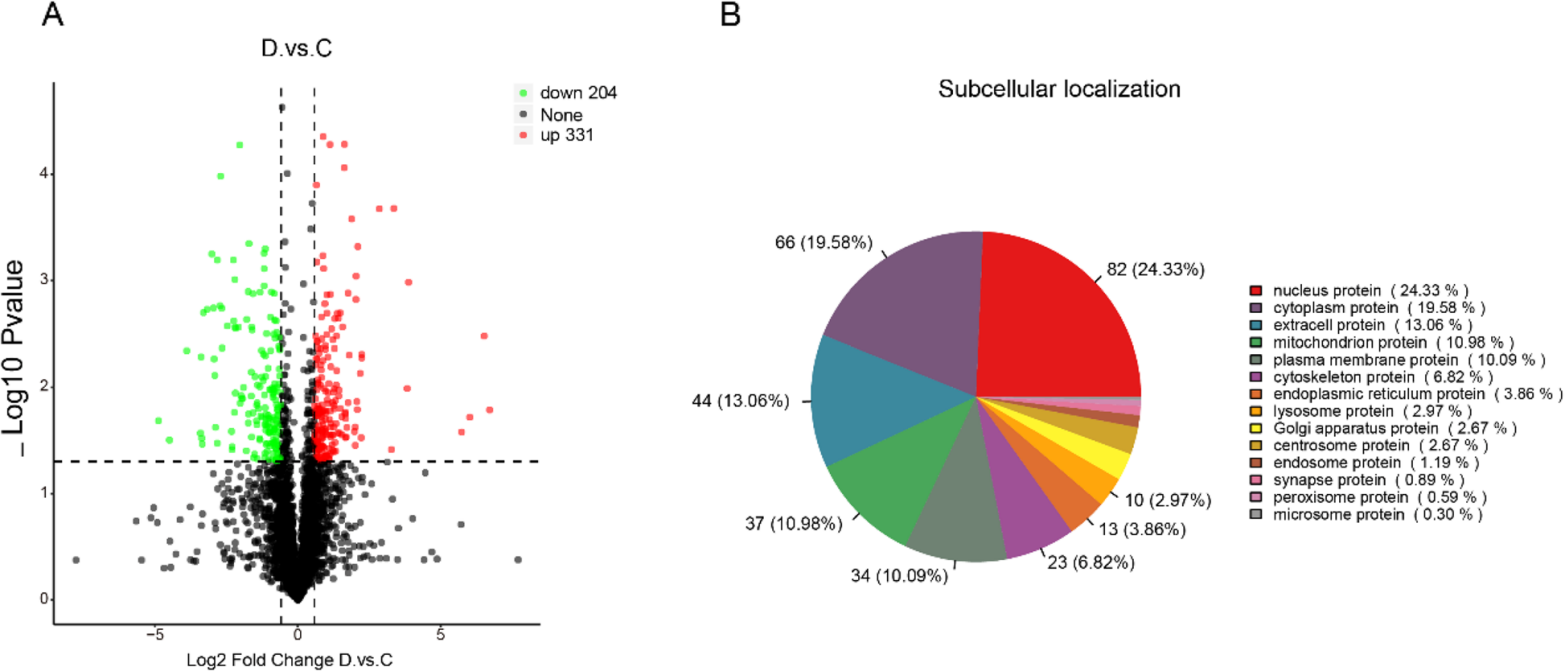
Overview of skin proteome data. **A** Differential protein expression in the skin of groups C and D. **B** Subcellular localization map of differential proteins

A total of 3,456 proteins were identified in tissues of group C and D, and a total of 535 differentially expressed proteins (DEPs), including 204 downregulated proteins and 331 upregulated proteins, were screened out using |Fold-change|> 1.5 and *p* < 0.05 as the threshold (Fig. 4A).

The DEPs were mainly nuclear proteins (24.33%), cytoplasmic proteins (19.58%), extracellular proteins (13.06%), mitochondrial proteins (10.98%), plasma membrane proteins (10.09%), and cytoskeletal proteins (6.82%) (Fig. 4B). To further identify scale formation-related proteins, the focus should be placed on extracellular proteins (epithelial extracellular proteins). KEGG enrichment analysis of differentially expressed proteins revealed that the DEPs were mainly enriched in Protein digestion and absorption, HIF-1 signalling pathway, and Relaxin signalling pathway (Figure S5).

### Combined transcriptomic and proteomic analysis

**Fig. 5:**
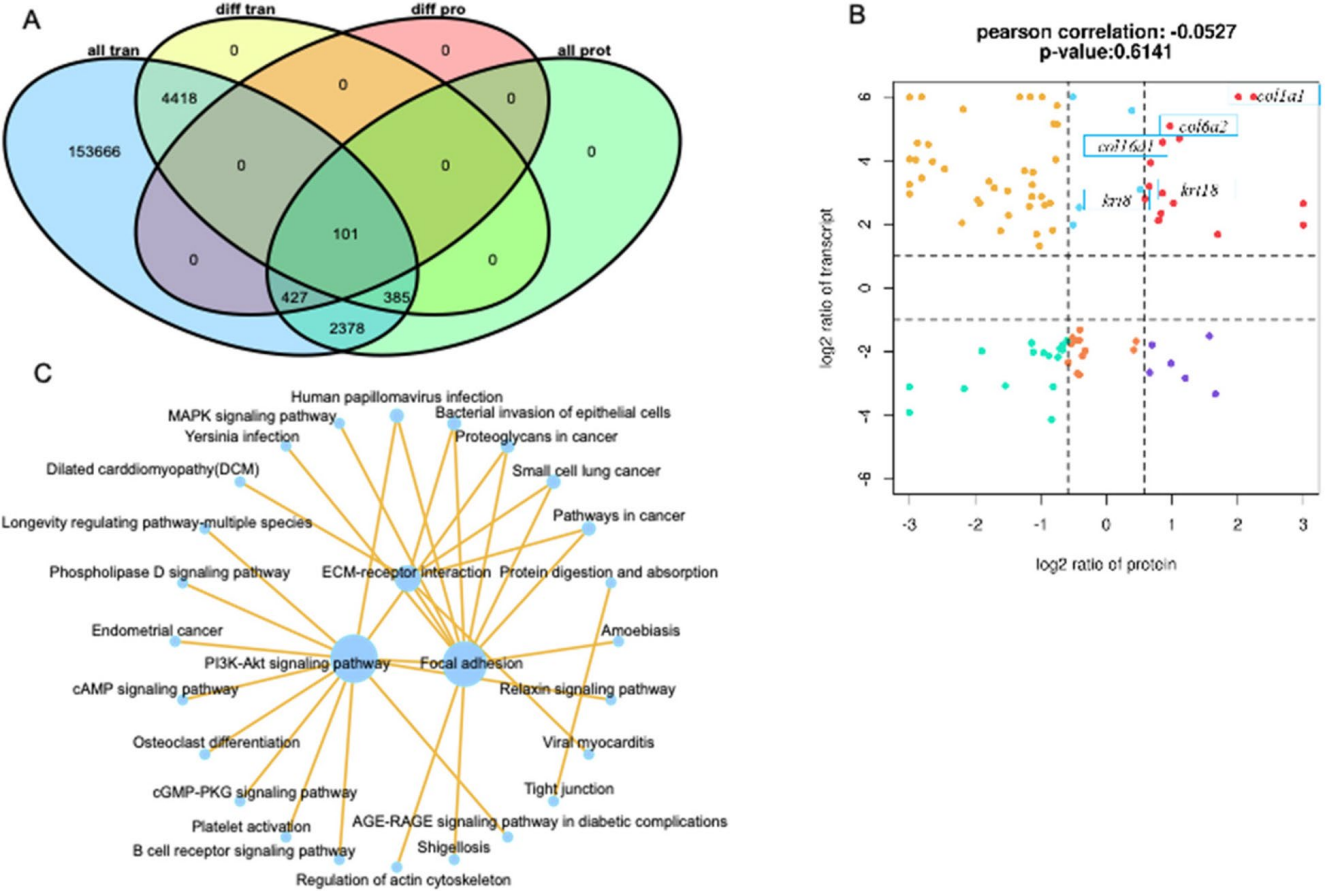
Combined analyses of skin transcriptome and proteome data. **A** Venn diagram of differential expressed genes and proteins. **B** Nine-quadrant map of transcriptomic and proteomic associations. Nine-quadrant maps based on transcriptomic and proteomic associations. **C** KEGG network diagram of the relationship of the core 30 signaling pathways

An association analysis between transcriptome (M vs. S) and proteome (C vs. D) was performed for the two datasets. According to the results shown in Fig. 5A, there are 3,242 genes (proteins) in common between the transcriptome and proteome, 101 of which are differentially expressed both at the mRNA and protein levels. In the nine-quadrant plot (Fig. 5B), 15 genes were found to be upregulated in scaled skin at both mRNA and protein levels (Table 3), including *col1a1*, *col6a2*, *col16a1*, *krt8*, *krt18* and so on. The proteins encoded by these genes are the most direct substances involved in the interaction between ECM and epithelial cells, and scales are skin appendages whose development was coordinately regulated by extracellular proteins and differentiation of epithelial cells.

**Table 3:** mRNA and protein both differentially significantly upregulated genes

ID	mrna_log2fc	pep_log2fc	*p* value	Description
Cluster-41776.49452	1.9794	3.364262	0.00023	XP_016096388.1 PREDICTED: apolipoprotein D-like [Sinocyclocheilus grahami]
Cluster-41776.84342	2.6644	1.022814	3.95E−09	XP_016357619.1 PREDICTED: CD81 protein-like [Sinocyclocheilus anshuiensis]
Cluster-41776.56022	3.9312	0.676582	4.24E−13	PREDICTED: four and a half LIM domains protein 2 [Sinocyclocheilus rhinocerous]
Cluster-41776.65243	7.5964	2.241662	3.53E−31	PREDICTED: collagen alpha-1..I.. chain-like [S. anshuiensis]
Cluster-41776.100456	3.1898	0.654239	0.000395	PREDICTED: macrophage-capping protein-like [S. rhinocerous]
Cluster-41776.63044	2.6506	3.83272	4.75E−05	PREDICTED: macrophage-capping protein-like [S. rhinocerous]
Cluster-41776.66755	5.08	0.971259	1.06E−23	XM_016508690.1 PREDICTED: S. rhinocerous thrombospondin-2-like (LOC107705413), mRNA
Cluster-41776.41509	2.1325	0.803643	0.000366	PREDICTED: EGF-containing fibulin-like extracellular matrix protein 2 [S. anshuiensis]
Cluster-41776.16031	4.6938	1.11047	5.53E−10	PREDICTED: cellular retinoic acid-binding protein 1 [Sinocyclocheilus]
Cluster-41776.83637	2.978	0.854641	8.54E−06	PREDICTED: keratin, type I cytoskeletal 18 [S. rhinocerous]
Cluster-41776.63465	2.8021	0.585165	2.83E−05	PREDICTED: keratin, type II cytoskeletal 8-like [S. grahami]
Cluster-41776.94807	1.6805	1.697574	0.000458	Hypothetical protein cypCar_00017267 [Cyprinus carpio]
Cluster-41776.3182	2.1172	0.790614	2.64E−05	Unnamed protein product, partial [Tetraodon nigroviridis]
Cluster-41776.59897	7.4265	2.013174	1.14E−25	PREDICTED: collagen alpha-1..I.. chain-like isoform X3 [Sinocyclocheilus]
Cluster-41776.80095	2.3457	0.831988	3.54E−05	PREDICTED: LOW QUALITY PROTEIN: keratin, type I cytoskeletal 18-like [Cyprinus]
Cluster-41776.53607	4.5753	0.858634	1.66E−27	PREDICTED: LOW QUALITY PROTEIN: keratin, type I cytoskeletal 18-like [Cyprinus]

To screen for key signaling pathways contributing to G. *przewalskii* scale formation, KEGG enrichment analysis was performed for these 101 DEGs and DEPs. 30 core signaling pathways were selected to analyze the interaction between different KEGGs. Based on the KEGG interaction network (Fig. 5C), it was hypothesized that the PI3K-AKT signaling pathway was critical for scale formation in *G. przewalskii*.

### Important signaling pathway and key gene association studies

**Fig. 6:**
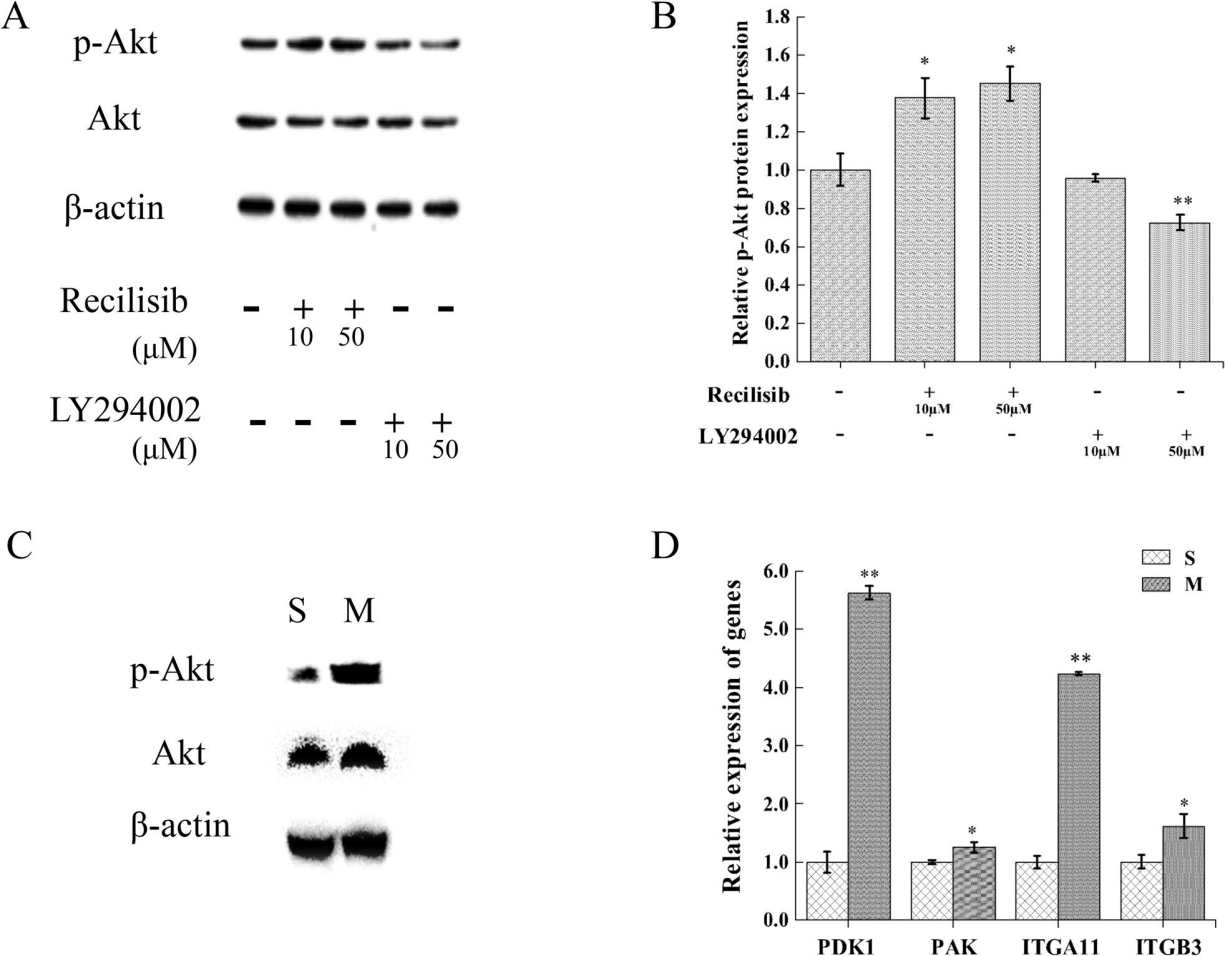
Expression analyses of key proteins and genes contributed to scale development in the PI3K-AKT signaling pathway. **A** Western blot analysis of Akt and p-Akt protein expression subjected to activator (Recilisib) or inhibitor (LY294002) treatment in *G. przewalskii* myofibroblast cell line. **B** Relative expression of p-Akt protein. Expression of p-Akt were determined by Image J and normalized to β-actin expression. p-Akt expression level was presented as fold increase relative to the control treatment (set to 1). * and ** indicated significant difference at *p* < 0.05 and* p* < 0.01 level, respetively. **C** Western blot analysis of p-Akt protein expression in skin tissues of the non-scaled dorsal area (S) and that around the scaled anal fin (M). **D** Relative expression of *PDK1*, *FAK*, *ITGA11*, and *ITGB3* genes in S and M tissues

The activation or inhibition of the PI3K-AKT signaling pathway is mainly determined by the phosphorylation of Akt molecules. With the use of the Qinghai Lake naked carp myofibroblasts [24] as a model, the PI3K-AKT signaling pathway activator (Recilisib) and inhibitor (LY294002) were added, and the expression levels of Akt, p-Akt, and other molecules were detected by western blot to determine the PI3K-AKT signaling pathway status. The results suggest that the p-Akt expression increased significantly after activator treatment, indicating that the PI3K-AKT signaling pathway was activated; in the group using the LY294002 inhibitor, p-Akt expression decreased significantly at 50 μM concentration, indicating inhibition of the PI3K-AKT signaling pathway (Fig. 6A, B). Therefore, it can be determined that the use of activators or inhibitors of signaling molecules can modify the state of specific signaling pathways and ensure the accuracy of the experimental results. Meanwhile, we investigated protein expression of both Akt and p-Akt in the scaled (M) and non-scaled (S) skin tissues by western blot. It was observed that the phosphorylation level of Akt (p-Akt) was significantly elevated in the scaled skin tissue, while no substantial change in expression observed for Akt (Fig. 6C). Gene expression of *PDK1*, *FAK*, *ITGA11*, and *ITGB3*, which constituted the PI3K-AKT pathway, was also significantly higher in the scaled skin tissue than that of the non-scaled (Fig. 6D), provided direct evidence for the involvement of PI3K-AKT pathway in scale development.

**Fig. 7:**
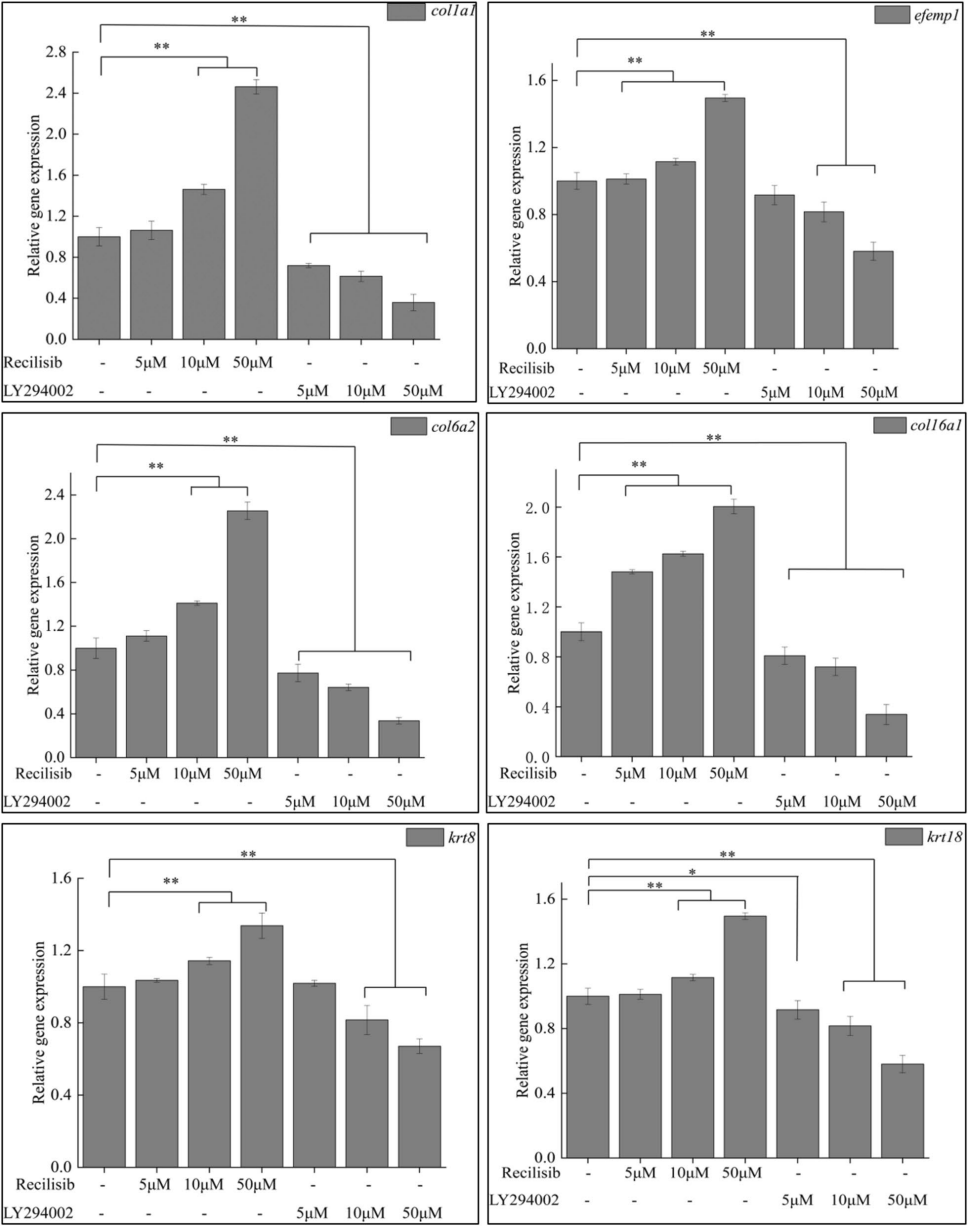
Relative expression of key genes subjected to Recilisib activator or LY294002 inhibitor treatment in the *G. przewalskii* myofibroblast cell line Note: "**" represents significant difference between the treatment group and the CK group at *p* < 0.01 level; "*" represents significant difference between the treatment group and the CK group at *p* < 0.05 level

A combined transcriptomic and proteomic analysis of the scaled and non-scaled skin tissues screened 15 important genes that were significantly upregulated both at the mRNA and the protein levels. Also, KEGG enrichment results indicated that the PI3K-AKT signaling pathway plays an important role in scale formation in *G. przewalskii*. Therefore, *G. przewalskii* myofibroblasts were treated with PI3K-AKT signaling pathway activator (Recilisib) and inhibitor (LY294002), and the relative expression of *emefp1*, *col1a1*, *col6a2*, *col16a1*, *krt8*, and *krt18* were detected by qPCR (Fig. 7) to investigate the regulatory relationship between the PI3K-AKT signaling pathway and genes (proteins) important for scale formation. The results indicated that the expression levels of *emefp1*, *col1a1*, *col6a2*, *col16a1*, *krt8*, and *krt18* were significantly upregulated when the PI3K-AKT signaling pathway was activated, and the expression levels of *emefp1*, *col1a1*, *col6a2*, *col16a1*, *krt8*, and *krt18* genes were significantly downregulated when the PI3K-AKT signaling pathway was inhibited.

## Discussion

Fish scale development is influenced by a variety of genetic and environmental factors, the regulatory mechanisms are very complex, and different fish scale development patterns are distinctive. The results of this study indicated that the initial developmental position of *G. przewalskii* scales was at the posterior edge of the gill cover, and this scale initiation position was the same as that reported for fishes of the family Cyprinidae, including grass carp (*C. idella*), common carp (*C. carpio*), heavy-mouthed cleavers (*S. davidi*), and floundering cleavers (*S. prenanti*) [5–7]. This differs from the onset of scale development in zebrafish, which starts at the lateral line of the caudal peduncle, and the same start of scale development is also observed in Nile tilapia (*Oreochromis niloticus*) [8]. Therefore, the fish scale coverage patterns varied significantly among species and were not very regular.

Mounting evidence has manifested that the cycloid scales of teleost fishes, including zebrafish, *G. przewalskii*, and goldfish, are all calcified tissues like bones that are abundant in osteoblasts, osteoclasts, and bone matrix proteins [25, 26]. In the present study, integrative analyses of transcriptome and proteome were performed, aiming to unveil key regulators controlling scale development in *G. przewalskii*. KEGG enrichment of DEGs revealed that protein digestion and absorption, ECM-receptor interaction, focal adhesion, PI3K-Akt signaling pathway, oxidative phosphorylation, AGE-RAGE signaling pathway in diabetic complications, platelet activation, axon guidance, fatty acid degradation, arachidonic acid metabolism, and TGF-beta signaling pathway were main pathways that potentially contributing to scale formation at significant level (q-value < 0.05). DEPs-based KEGG enrichment indicated that protein digestion and absorption, HIF-1 signaling pathway, relaxing signaling pathway, ribosome, tyrosine metabolism, lysosome, bladder cancer, AGE-RAGE signaling pathway in diabetic complications, aldosterone synthesis and secretion, adrenergic signaling in cardiomyocytes were pathways that significantly enriched at *p* < 0.05 level.

Ranking by the q-value, protein digestion and absorption was the most significantly enriched pathway in DEGs-based analysis. Detailed analysis of gene composition unveiled that collagen-related genes, including 1A, 2A, 4A, 5A, 6A, 7A, 9A, 10A, 11A, 12A, 15A, 16A, 17A, 18A, 21A, 22A, 24A and 27A, occupied a large proportion (80%) of the genes enriched in the protein digestion and absorption pathway. Reappearance of this pathway in DEPs-based KEGG enrichment led us to confirm that collagen-related genes were one of the key regulators controlling scale development [27], though the corresponding proteins involved were confined to 9 (1A, 2A, 4A, 5A, 6A, 7A, 11A, 16A, 18A). The collagen-related genes consist 28 members [28], the majority of which were identified in the transcriptome analysis of this study. The higher expression levels of collagen-related genes in the scaled tissue (around the anal fin) reflected the significance of collagen-related proteins for scale formation in *G. przewalskii*.

In teeth tissues, collagen is an essential component of the ECM [29]. The characteristic banding pattern formed by type I collagen was supposed to guide calcium phosphate deposition in dentin matrix [30]. This echoed our finding that ECM was the second most significantly enriched pathway in DEPs-based KEGG enrichment. Dissection of the ECM pathway revealed that laminin and fibronectin were also involved, both of which are promoters of ECM biomineralization [31]. Focal adhesion was also found a major pathway that might related to scale formation in *G. przewalskii*. The contribution of focal adhesion pathway to biomineralization, as well as elevated expressions of the β-actin, actinin, FAK genes suggesting the progressing biomineralization in *G. przewalskii* scales [32]. Oxidative phosphorylation was identified as a KEGG that significant enriched in the DEGs-based pathway analysis. The oxidative phosphorylation pathway encompasses genes that proved as involvers of energy metabolism and biomineralization, such as cytochrome c oxidase, NADH dehydrogenase, cytochrome c reductase, etc. Expression profile revealed that 8 out 17 members of the cytochrome c oxidase family and 16 out 38 members of the NADH dehydrogenase family were down-regulated in the scaled skin tissue of *G. przewalskii*. This suggested that non-scaled skin regions might play an important role in fish respiration. The high expression of cytochrome c oxidase and NADH dehydrogenase family genes corresponds well to the transcript level of mitochondrial genes. In macrophage cells, dysfunction of cytochrome c oxidase genes led to enhanced osteoclast formation [33]. Previous studies demonstrated that both cytochrome c oxidase and NADH dehydrogenase showed higher expression levels in the mineralized regions than that of the non-mineralized regions [34, 35]. Thereby, the involvement of cytochrome c oxidase and NADH dehydrogenase genes in biomineralization still need further confirmation.

The “AGE-RAGE signalling in diabetic complications” pathway, which partially overlapped with the TGF-β signaling, was also one of the most significant KEGG pathway enriched in *G. przewalskii*. Both these pathways have been shown to be involved in the biomineralization process [36]. The PKC gene of the “AGE-RAGE signalling in diabetic complications” pathway was identified as a regulator of biomineralization. As a key participant of the Ca^2+^ signalling, PKC might exert influence over biomineralization by regulating calcium-related biological processes. In addition, PKC has been proved to influence biomineralization through AP1, a common regulator of VEGF and MMP2. The role of AP1 in the biomineralization process of pearl oyster has been demonstrated in our previous work [37]. VEGF functions as a differentiation inducer of osteoblasts, and has been verified as a critical regulator of bone formation in mammals [38]. The MMP-2, whose expression was detected in zebrafish osteoblasts, was found as a crucial regulator of scale regeneration [39]. The “AGE-RAGE signalling in diabetic complications” pathway was also significantly enriched in DEPs-based KEGG analysis.

Proteins of the axon guidance pathway, mainly semaphorins and ephrins, are also identified as biomineralization regulators. Semaphorins constitute a large family of membrane-associated and secreted proteins. Sema3A, whose mutation resulted skeletal abnormalities in mice, was the first mineralization-related protein identified in Semaphorin family [40]. In the present study, elevated expression of sema3A and sema5 was observed in the scaled tissue of *G. przewalskii*, suggesting the involvement of Semaphorins in scale development. In light that sema5-controlled biomineralization is rarely reported, sema5 might be a novel regulator of biomineralization in *G. przewalskii*. Sema4D and sema6, whose interaction partners were Plexin-B1 and Plexin-A1 respectively, were also found to be involved in bone metabolism [41]. However, significant changes in expression were not detected for sema4D and sema6 in our comparative analysis. It is speculated that semaphorins might share similar functions during the biomineralization process in *G. przewalskii*, though the paralogues involved is different.

The critical role of Ephrin–Eph signaling in skeletal system development has been identified in previous report [41]. The Eph family receptors which are subdivided into two groups, i.e. EphA and EphB, contains 14 members in sum. Ephrins, the ligands for Ephs, comprise two subclasses, including type A (glycophosphatidylinositol (GPI)-anchored membrane-bound type) and type B (transmembrane type). In our case, elevated expression of four Eph receptors (EphA4, EphA5, EphB2, EphB3), as well as three Ephrin ligands (EphrinA2, EphrinA5, EphrinA5b) was observed in the scaled skin tissue of *G. przewalskii*. But enhanced expression of type B ephrins were not detected. Hence, it is speculated that EphrinB-related signallings (such as EphrinB2-EphB4 and EphrinB1-EphB2), which were established as essential regulators of bone homeostasis [41], might play a limited role in *G. przewalskii* scale biomineralization. The regulatory effects of the four Eph receptors (EphA4, EphA5, EphB2, EphB3) and three Ephrin ligands (EphrinA2, EphrinA5, EphrinA5b) on mineralization are still awaited to be verified.

Fatty acid degradation was identified as the third most significantly enriched DEGs-based KEGG pathway in the present study. It has been manifested that fatty acid and related metabolites could modulate bone metabolism through multiple ways, including inflammation, oxidative stress, autophagy and apoptosis [42]. The “arachidonic acid metabolism” pathway was also found to be significantly enriched in scale formation of *G. przewalskii*. During generation of arachidonic acid, PLA2G4 proteins (especially PLA2G4A) promote arachidonic acid synthesis by selective hydrolyzation. Arachidonic acid derivatives, PGI-2 and PGE-2, were found to suppress differentiation of osteoblasts through their inhibitory effects on TGF-β and RUNX2 expression [43]. The platelet activation pathway, which is intertangled with the ECM signaling and the arachidonic acid metabolism pathways, is also considered as a signaling implicated in biomineralization.

Compared to KEGG pathways enriched in transcriptome analysis, KEGGs resulted from DEPs enrichment were much less. The hypoxia-inducible factor (HIF) signaling pathways, relaxin, adrenergic signaling, and ribosome were main KEGG pathways that might function as scale-genesis regulators. The hypoxia-inducible factor (HIF) signaling pathways has been found to be involved osteogenesis. The HIF-1 transcription factor is a potent regulator of the expression of VEGF, PDK1, and EPO [44, 45]. The augmented effects of relaxin on bone formation is relied on BMP-2, and relaxin treatment could stimulate the differentiation of mesenchymal stem cells into osteoblasts [46, 47].

The ribosome pathway is also identified as a regulator of osteoblast differentiation. RUNX2 and FGFR2 were found to influence transcription of rRNA genes, which built a potential evidence link between ribosome biogenesis and osteogenesis [48]. But studies concerning ribosome-involved biomineralization are rare. A total of 17 ribosomal proteins (16 up-regulated and 1 down-regulated) were detected to be differentially expressed in *G. przewalskii* when compared the scaled skin tissue with that of the non-scaled. The role of ribosome proteins in scale genesis still need to be clarified with more direct evidence.

Proteins of the lysosome pathway, including AP-1, AP-3, cathepsin K, and cathepsin D, are all found involved in osteogenesis-related signalings. Interaction of ARAP1 with the AP-3 adaptor complexes facilitated the bone-digesting process in osteoclasts [49]. Cathepsin K (CatK), a potent protease, was found to mediate bone resorption [50]. As highly calcified tissues, scales store massive amount of calcium that can be easily absorbed by the fish body (up to 20% of the total calcium) [39]. The high expressions of AP-3 and CatK suggesting that these two genes might serve as regulators balancing body calcium need and scale development (just like the skeleton in mammals). Cathepsin D has been identified as a mineralization inhibitor [51], whose expression was significantly higher in the non-scaled skin tissue in *G. przewalskii*. Combining expressions of AP-1 (up-regulated in scaled skin tissue), AP-3 (up-regulated in scaled skin tissue), cathepsin K (up-regulated in scaled skin tissue), and cathepsin D (up-regulated in non-scaled skin tissue), the results of our proteome data basically match their function in osteogenesis of previous reports.

In bone marrow, the adrenergic signaling drives the enhancement of the vitamin D receptor (VDR) signaling, which in turn suppresses the activity of the osteoblast cells [52]. As a key protein of the adrenergic signaling pathway, tropomyosin mediate interactions between actin and other proteins. Since expression of the actin genes was elevated during the scale formation *G. przewalskii*, it is reasonable to speculate that tropomyosin might play vital roles in scale formation through mediating protein interactions (for instance the interaction between actin and myosin). The serine/threonine-protein phosphatase 2A (PP2A) family proteins were identified as regulators of osteoblast cells. Differentiation of osteoblast progenitor cells, proliferation of osteoblasts, and metastasis of osteosarcoma cells were all tightly related to proteins of the PP2A family [53]. Studying the function of PP2As in *G. przewalskii* scale development could facilitate the understanding of their roles in scale genesis of fish species. Proteins of the extracellular signal-regulated kinase (ERK) family, which belong to the broad mitogen-activated protein kinase family, were also found to regulate osteogenesis. ERKs were identified as interacting partners of a plethora of osteogenesis-related proteins, including RUNX2. In addition, interactions between ERKs and other parallel signaling pathways were also detected in osteoblasts (for instance the Wnt/β-catenin pathway) [54]. These promote us to infer that ERKs might also key regulators of the scale genesis biological process.

Proteins of the bladder cancer pathway, i.e. ERK and RAF, are also regulators of osteogenesis. In hypertrophic chondrocytes, Raf kinases are essential for the phosphate-induced phosphorylation of ERK1/2 [55]. As to tyrosine metabolism and aldosterone synthesis and secretion pathways, there have been few reports connecting these two pathways to biomineralization, osteogenesis, or scale genesis. Their roles and related functions in scale development still need to be explored.

In the present study, the expression levels of the DEGs and the corresponding DEPs do not correlate well. This discordance has been noticed by earlier studies [56] and the study results of our own [24]. The low correlation between transcriptome and proteome data was ascribed to several reasons, including low abundance of transcripts or proteins (challenging to detect accurately), poor protein recovery (low solubility or attachment to membranes) [57], translation efficiency, mRNA expression variability, post-transcriptional modification, protein turnover and developmental stages [58–61]. Besides, due to measurement errors, the imperfect transcriptome-proteome correlations could also be attributed to technical reasons [57]. It is important to highlight that discrepancies between RNA-seq and proteome sequencing results have been consistently observed and confirmed in various studies involving microorganisms [57, 61], plants [60, 62], and animals [63, 64]. In our recent work, the expression level of more than 86% of the DEGs did not correlate well the abundance of the corresponding proteins in *G. przewalskii* under saline-alkaline stress treatment.

Transcriptomic analysis of the non-scaled dorsal skin tissue and that around the anal fin revealed that 4,904 DEGs existed between these two comparison groups. The DEGs comprised members of different gene families such as EDAR, Wnt10, FGF, and BMP. The EDA pathway is also found in zebrafish and medaka (Oryzias latipes), where it is involved in the development of scales, teeth, and fins [65–68]. Mutations in this pathway can also lead to abnormal scale development, characterized by the partial or complete absence of scales [69]. There was a significant difference in the expression of the EDAR gene in the scaled growth and non-scaled skin tissues of *G. przewalskii*, suggesting a role in scale formation. The results also indicated that genes such as wnt1, wnt1, wnt4, wnt5, and Fzd1 were differentially expressed in scaled growth and non-scaled sites, all of which were associated with the Wnt signaling pathway, suggesting that this signaling pathway plays an important role in scale formation in *G. przewalskii*. Many studies have suggested that Wnt/β-catenin signaling regulates the development of hair follicles [70–72]. Studies have indicated that it plays an important role in the early stages of scale development and interacts with EDA signaling during scale development in zebrafish [73]. The role of FGF in the development of skin accessory organs has also been reported [74–76]. For example, in zebrafish scale development, mutations in FGFR1a and FGFR20a result in modifications in the size of zebrafish scales [77]. In our study, we found that expressions of FGF1, FGFR1, FGF2, and FGFR2 differed in the two groups, confirming that the FGF pathway plays an important role in the formation of scales in *G. przewalskii*. BMP is a bone morphogenetic protein, and the BMP/BMPR signaling pathway in zebrafish is involved in fin development [78], and it was later indicated to be involved in the biomineralization process [79]. In this study, transcriptome data showed that BMP gene family members bmp2, bmp7, bmp8, and bmpr2 were significantly upregulated in scaled skin tissue.

Among the top-16 DEGs-based KEGG pathways enriched, the PI3K-AKT signaling was identified as the pathway with the largest number of genes clustered (125 genes), suggesting its strong connection with scale formation. Subsequent integrative analysis facilitated identification of the 15 gene-protein pairs (efemp1, col16a1, col6a2, col1a1, krt8, and krt18) that positively correlated. The relationship between the PI3K-AKT signaling pathway and important genes was verified by myofibroblast cell lines. We found that when the PI3K-AKT signaling pathway was blocked with a signaling pathway inhibitor, col1a1 expression levels decreased, and when a small molecule activator of the PI3K-AKT signaling pathway was used, col1a1 expression levels increased accordingly, suggesting that the PI3K-AKT signaling pathway can positively regulate col1a1 expression. These results are consistent with those of previous reports that a new clinical-stage activator of inositol phosphatase-1 (SHIP1) can promote the expression of osteoblast-related genes, including col1a1 and osteocalcin, by upregulating the phosphorylation levels of PI3K and AKT [80]. Collagen col16a1 is an osteoblast marker, and it has been used as a key gene for research during bone development [81]. Activation of the PI3K-AKT signaling pathway significantly upregulated the expression of osteogenic genes [82–84]. It has also been reported that inhibit of β-catenin transcriptional activity attenuated PI3K/AKT-induced osteoblast proliferation, differentiation, and mineralization, and the PI3K/AKT pathway was also activated by and involved in Wnt3a-induced osteoblast proliferation and differentiation. The process of scale formation and bone development involves biomineralization, suggesting complex crosstalk of the PI3K-AKT signaling pathway with both the Wnt and the TGF-β signaling pathway. Combining the results of histological data analysis and cellular experiments, we concluded that the PI3K-AKT signaling pathway plays a crucial role in scale formation in *G. przewalskii*. However, the crosstalk between the PI3K-AKT signaling pathway, Wnt signaling pathway, and TGF-β signaling pathway needs to be further investigated.

## Conclusion

Transcriptomic and proteomic data were combined to screen key genes and important signaling pathways in the development of *G. przewalskii* scales and to provide reference data for the development of skin appendages in other animals. The transcriptomic and proteomic data analysis results showed that differentially expressed genes were significantly enriched in the PI3K-AKT signaling pathway. The expression of *col1a1*, *col6a2*, *col16a1*, *emefp1*, *krt8*, and *krt18* genes, as well as their corresponding proteins, was significantly higher in the scaled skin sites compared to the scale-free sites, and the reliability of transcriptomic data was verified by qPCR. The primary components of fish scales are hydroxyapatite and collagen. Therefore, the expression of collagen and keratin is closely related to scale formation. Meanwhile, to verify whether important signaling pathways have regulatory relationships with key genes, fibroblast cell lines were used as a model to activate and block the PI3K-AKT signaling pathway using small molecule activators and inhibitors of the signaling pathway, and the results showed that the PI3K-AKT signaling pathway can positively regulate *emefp1*, *col1a1*, *col6a2*, *col16a1*, *krt8*, and *krt18* expression, and the activation and blockage of the signaling pathway were verified using western blot analysis. The results of this study indicate that the developmental process of *G. przewalskii* scales is regulated by multiple genes and signaling pathways. Thus our study revealed some of the regulatory mechanisms of *G. przewalskii* scale development, especially signaling mediated by the PI3K-AKT pathway.

## Methods

### Animals

The fish needed for the experiments were provided by The Rescue and Rehabilitation Center of Naked Carps in Lake Qinghai (Xining, China). For scale structure observation, three fish with normal development and healthy body condition, 25.4 ± 1.5 cm in length and 27 ± 1.2 g in weight, were selected and euthanized with a lethal dose (80.0 mg/L) of MS-222 (Sigma, Shanghai, China). The entire skin behind the gills and the anal fins of the fish were obtained, soaked in 5% NaOH solution to remove mucus, and repeatedly rinsed with deionized water to obtain clean scales, after which they were naturally dried, sprayed with gold, and photographed for scanning electron microscopy observation.

For the observation of scale coverage pattern during development, juveniles of 60-days post-hatch were selected every 2 days, and the fish samples were first placed in 4% paraformaldehyde for 24 h. Then the posterior gill skin and rump skin were obtained intactly, dehydrated, stained with 2% Alizarin Red S, decolored, and observed under a stereomicroscope (Leica M205, Germany).

For preparation of transcriptomic and proteomic sequencing samples, three healthy 2-year-old juvenile fish, 20.5 ± 1.4 cm in length and 24 ± 0.8 g in weight, were selected and euthanized with a lethal dose (80.0 mg/L) of MS-222 (Sigma, Shanghai, China). The dorsal skin tissue (without scales) was dissected from each fish, washed with 1 × phosphate buffered solution (PBS), and then quickly placed in 2-mL lyophilization tubes for liquid nitrogen freezing; the samples for the transcriptome group were recorded as group S, and those for the protein group were recorded as group D. The skin tissue (with scales) around the anal fin was also obtained, washed with 1 × PBS, and then quickly placed in 2-mL lyophilization tubes for liquid nitrogen freezing; the samples for the transcriptome group were recorded as group M, and those for the protein group were recorded as group C. Subsequently, transcriptomic and proteomic experiments were performed. Three biological replicates were set up for each group: M1, M2, and M3; D1, D2, and D3; S1, S2, and S3; and C1, C2, and C3.

### Library preparation, Illumina sequencing

For sample preparation of RNA sequencing, a total of 1.5 µg RNA was used as the input material for each sample. Construction of sequencing libraries was performed using the NEBNext® Ultra™ RNA Library Prep Kit (NEB, USA), following instructions of the manufacturer. Enrichment of mRNA were carried with poly-T attached magnetic beads. After fragmentation, mRNA samples were subjected to first-strand cDNA synthesis by using random hexamer primer and M-MuLV reverse transcriptase (RNase H-). Subsequent synthesis of the second strand cDNA was performed with DNA polymerase I and RNase H. The remaining sequence overhangs were then blunted by exonuclease/polymerase activity. After adenylation of the 3’ end, DNA fragments were ligated to NEBNext adaptors. For preferential selection of fragments (250 ~ 300 bp), cDNAs were purified by AMPure XP system (Beckman Coulter, Beverly, USA). Then cDNAs fragments with aptamer-linked were selectively enriched by PCR. Lastly, purification of PCR products was performed by the AMPure XP system, quality of the library constructed was evaluated using the Agilent Bioanalyzer 2100 system. According to the manufacturer's instructions, we used TruSeq PE Cluster Kit v3-cBot-HS (Illumina) to cluster the index-coded samples on a cBot Cluster Generation system. Sequencing was performed on an Illumina Hiseq platform by Novogene Co., Limited.

### Data processing and transcriptome assembly

Raw data was quality-filtered with the Trimmomatic software in order to remove adaptors and low-quality bases (parameters: ILLUMINACLIP: adapter. fasta: 2: 30: 8 LEADING: 3 TRAILING: 3 SLIDINGWINDOW: 4: 15 MINLEN: 40). Statistics of the sequencing data, including Q20, Q30 and GC content, were then calculated. Subsequent analyses were all based on the clean data generated. The clean reads were then assembled into expressed sequence tag clusters (contigs) and de novo assembled into the transcript by using Trinity [85], with min_kmer_cov set to 2 and all other parameters set default.

### Gene function annotation and differential analyses

Unigenes detected by high-throughput sequencing were subjected to gene annotation in seven databases: Nr, Nt, Pfam, KOG, Swiss-Prot, KEGG, and GO. For annotation against NR, KOG/COG, and Swiss-Prot databases, diamond v0.8.22 was used. Nt annotation was performed with the NCBI blast 2.2.28 + . For annotation with the KEGG, Pfam, and GO databases, KAAS (r140224), hmmscan (HMMER 3), and blast2go (b2g4pipe_v2.5) were used, respetively. All the parametic details related to the software packages used were listed in Table S6. For quantification of gene expression, RSEM was used. The clean data were mapped back to the assembled transcriptome by Bowtie2 (v1.2.15) with default settings (mismatch = 0). For identification of differentially expressed genes, read count for each gene was compared using DEseq v1.10.1 with screening threshold of *p*-adjust < 0.05 and |log2Fold-Change|> 1.

### GO enrichment analysis and KEGG pathway enrichment analysis

Gene Ontology (GO) enrichment analysis of the identified DEGs was implemented by the GOseq R packages based on the Wallenius non-central hyper-geometric distribution [86]. The KOBAS tool (v2.0.12) was utilized for the KEGG enrichment analysis of DEGs, and Benjamini–Hochberg multiple testing adjustment was applied for *p*-value correction [87].

### qPCR analysis

The RNA of each sample was extracted according to the instructions of Trizol extraction, and then Primer 3.0 online software was used to design upstream and downstream primers (Table S7) for fluorescence quantification according to the full-length sequences obtained from cloning. The total RNA of each tissue was reverse transcribed according to the instructions of PrimeScript™ RT reagent Kit with gDNA Eraser (Perfect real time) to obtain cDNA for fluorescence quantification. Subsequent qPCR was performed according to the instructions of the TB Green® Premix Ex Taq™ II (Tli RNaseH Plus) kit (with a 20 μL reaction system). Configuration of the reaction mixture was as follows: Forward Primer 0.8 μL, Reverse Primer (10 µM) 0.8 μL, TB Green® Premix Ex Taq™ II 10.0 μL, ddH_2_O 6.4 μL, and cDNA 2.0 μL. Reaction Procedure: 95 °C, 30 s; 95 °C, 5 S; 55 °C, 30 s, 72 °C, 30 s for 40 cycles, and 3 replicates for each sample. After data export, the results were analyzed using the 2^−ΔΔct^ method.

### Protein isolation, digestion and proteome analyses

Proteome analysis of the scaled and non-scaled skin tissues was carried out to identify proteins that potentially involved in scale development. In brief, samples were first subjected to protein extraction using the cold acetone method. Concentration of the total protein was then determined by using the BCA method. Quality of the protein sample was further checked by SDS-PAGE. After quality check, proteins were then fully digested with trypsin. Digested samples were centrifuged, lyophilized, and reconstituted in TEAB. The peptides were then labeled with TMT-10 tags, following instructions of the manufacturer. Labeled peptides were then subjected to fractionation by high-pH reversed-phase (RP) separation. The collected peptide fractions were analyzed by an Easy-nLC 1200 UHPLC system (ThermoFisher Scientific, USA). Proteome analysis was performed with a Q Exactive HF-X mass spectrometer (ThermoFisher Scientific, USA). The resulting data were subjected to identification and quantification with the Proteome Discoverer 2.2 software. The filtering thresholds |Fold-change|> 1.5 and *p* < 0.05 (T-test) was applied for DEPs screening.

### Transcriptomic and proteomic association analyses

Genes that were differentially expressed in the transcriptomic data were correlated to the proteomic data to further identify candidates that critical for scale development at both the mRNA and protein levels. Pearson’s correlation coefficients (R) and corresponding *p*-values were calculated to screen significant correlations using a nine-quadrant map.

### Validation of the PI3K-AKT pathway in the regulation of candidate genes for scale development

Stably passaged *G. przewalskii* myofibroblast cell line [24], was equally divided into the cell culture of 6-well plates, after which the cells were cultured for 2 d so that the number of cells per well was roughly 2–4 × 10^6^. Cell starvation treatment was then performed for 12 h. The inhibitor (LY294002, Cat. No. HY-10108) or the activator (Recilisib, Cat. No. HY-101625) of the PI3K-AKT signalling pathway, was added to the cell culture, respectively. Three concentration gradients, i.e. 5 μM, 10 μM, and 50 μM, were set up for both the inhibitor and the activator treatments. Cells without inhibitor and activator treatments served as the control. After 24 h, the treated cells were collected and subjected for RNA extraction. The primers used for qPCR quantification are presented in Table S7.

### Western blot

Total protein from cells and tissues–the skin of S (dorsal area without scale) and M (area around the anal fin with scales) was obtained using the ProteoPrep® Total Extration Sample Kit (Sigma-aldrich, USA). Protein concentration was determined using the BCA Protein Assay Kit (Solarbio, China). Samples were quantitatively diluted to a concentration of 10 μg/μL, and adds of 7 μL were run on SurePAGE (M00668, GenScript, USA) at 120 V for 60 min, followed by wet transfer to polyvinylidene fluoride (PVDF) membranes (Merck, USA). The PVDF membranes were blocked at room temperature for 2 h after electrotransfer with a blocking solution (TBST + 5% skim milk powder). Then, the membranes were incubated with AKT protein antibody as the primary antibody (#4691; Cell Signaling Technology) at a dilution ratio of 1:1,000, P-AKT protein antibody (66,444–1-Ig; ProteinTech) at a dilution ratio of 1:4,000, and internal doped β-actin antibody (MA1-140; Invitrogen) at a dilution ratio of 1:10,000, followed by incubation at 4 °C for 12 h, and the membranes were washed with PBST for 15 min each time for a total of four times. Afterward, the membranes were incubated with horseradish peroxidase (HRP)-conjugated diluted (1:2,000) secondary antibody for 1 h at room temperature and washed with PBST for a total of four times for 15 min. The proteins were visualized with a chemiluminescent gel imaging system.

### Statistical analysis

Origin Pro 2010 (OriginLab, USA) was used to analyze and plot the data. Data are expressed as the mean ± standard error of three replicates.

### Supplementary Information

**Additional file 1.**

**Additional file 2.**

**Additional file 3.**

**Additional file 4.**

**Additional file 5.**

**Additional file 6.**

**Additional file 7.**

## Data Availability

The mass spectrometry proteomics data have been deposited to the ProteomeXchange Consortium (https://proteomecentral.proteomexchange.org/cgi/GetDataset?ID=PXD042214) via the iProX partner repository [88, 89] with the dataset identifier PXD042214; Transcriptome data are currently being uploaded to National Genomics Data Center GSA Systems [GSA: CRA010997].
