# Supplementary figures and images for: Transcriptomic and proteomic strategies to reveal the mechanism of Gymnocypris przewalskii scale development
Source: BMC Genomics. 2024 Feb 3;25:140. doi: 10.1186/s12864-024-10047-1 (PMC10837935; doi:10.1186/s12864-024-10047-1)

Enriched GO Terms  
(MvsS\_down)

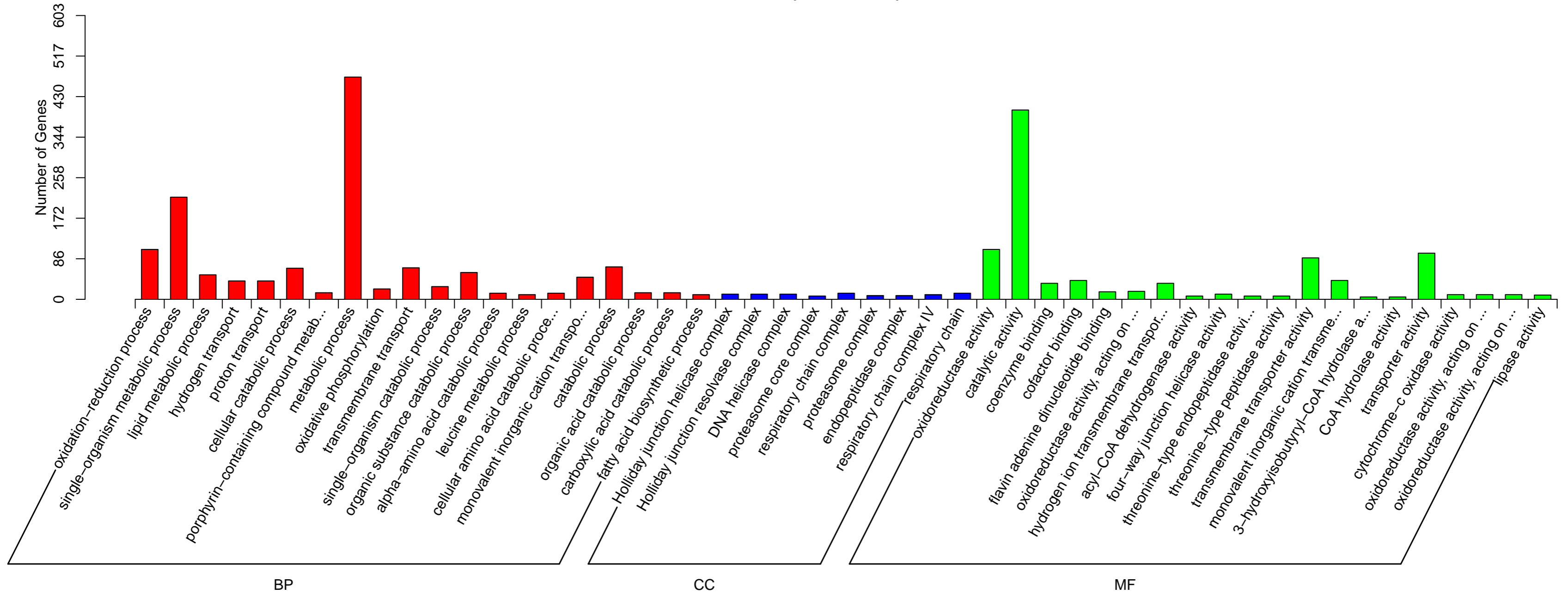

Supplement: Supplementary file 1 — Additional file 1. [file 12864_2024_10047_MOESM1_ESM.pdf]

Enriched GO Terms  
(MvsS\_up)

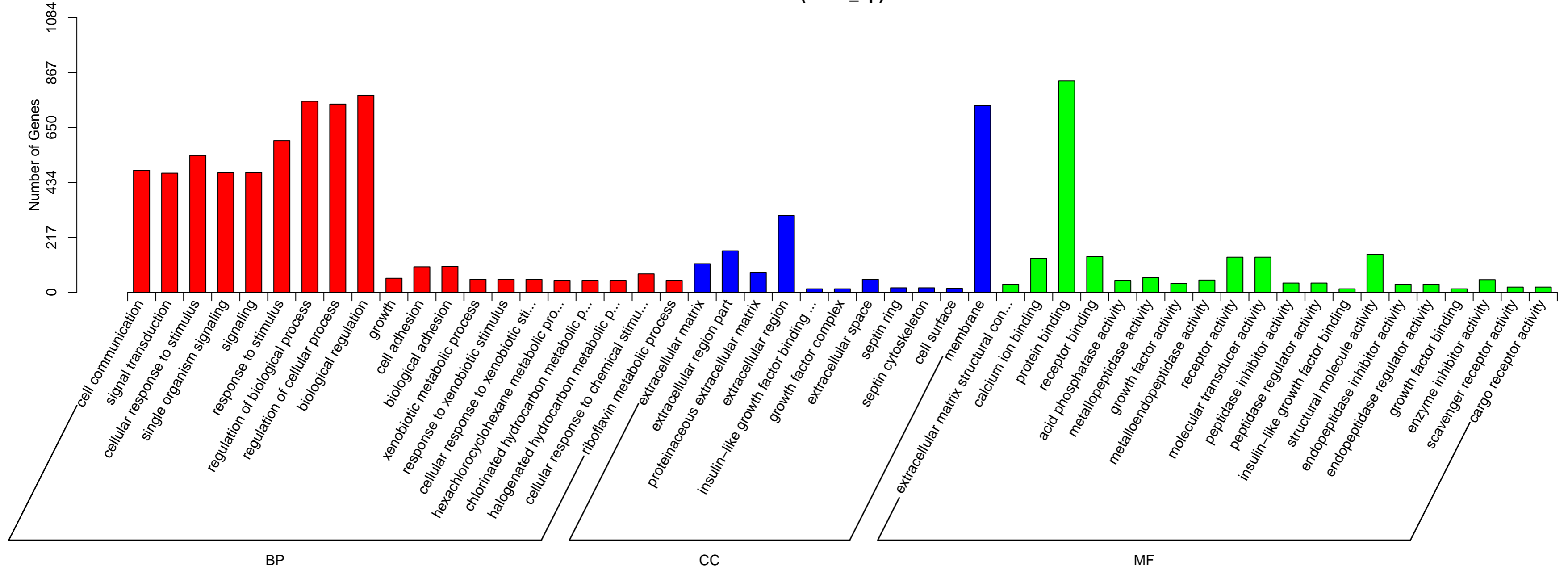

Supplement: Supplementary file 2 — Additional file 2. [file 12864_2024_10047_MOESM2_ESM.pdf]

# Statistics of Pathway Enrichment

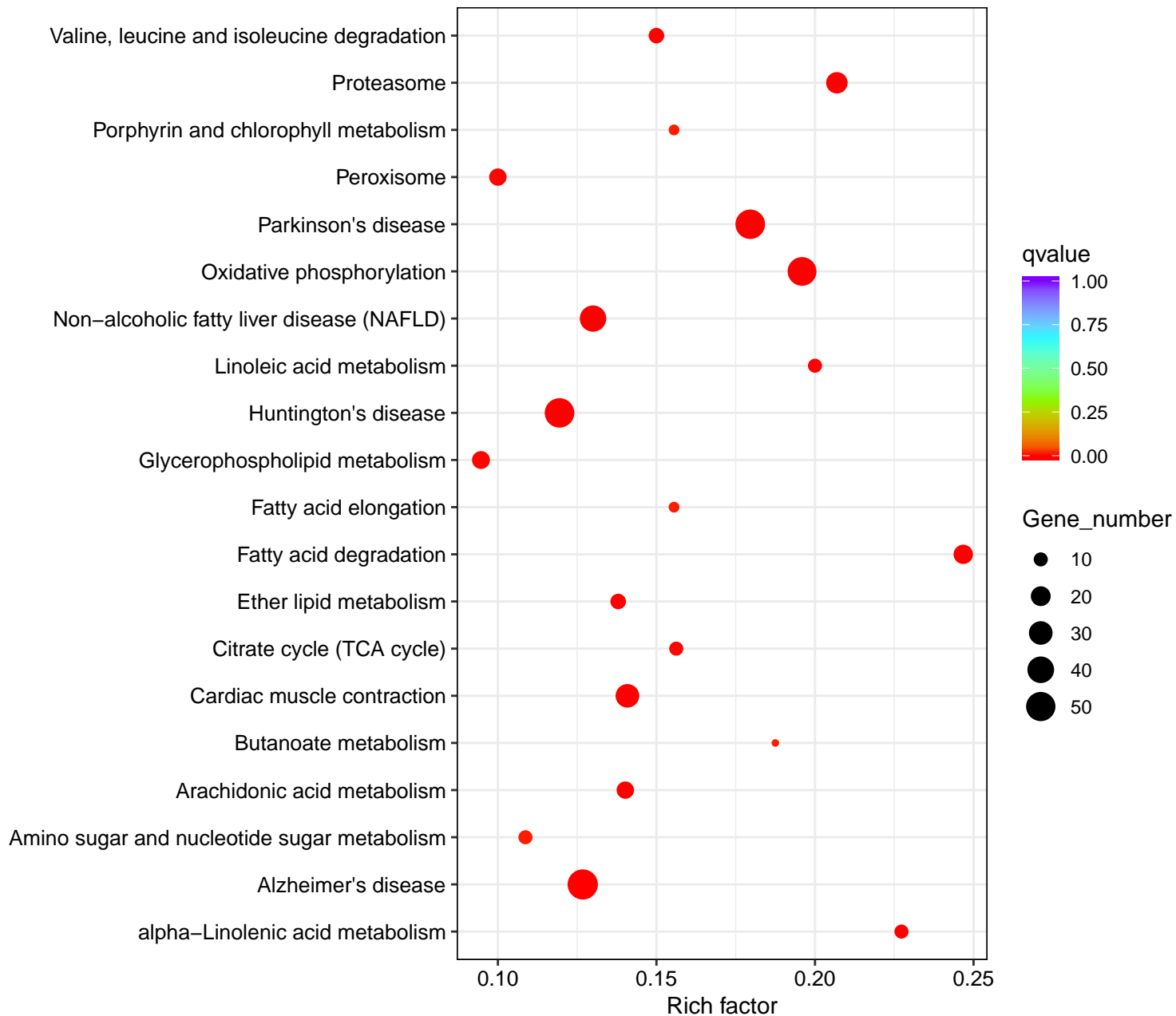

Supplement: Supplementary file 3 — Additional file 3. [file 12864_2024_10047_MOESM3_ESM.pdf]

# Statistics of Pathway Enrichment

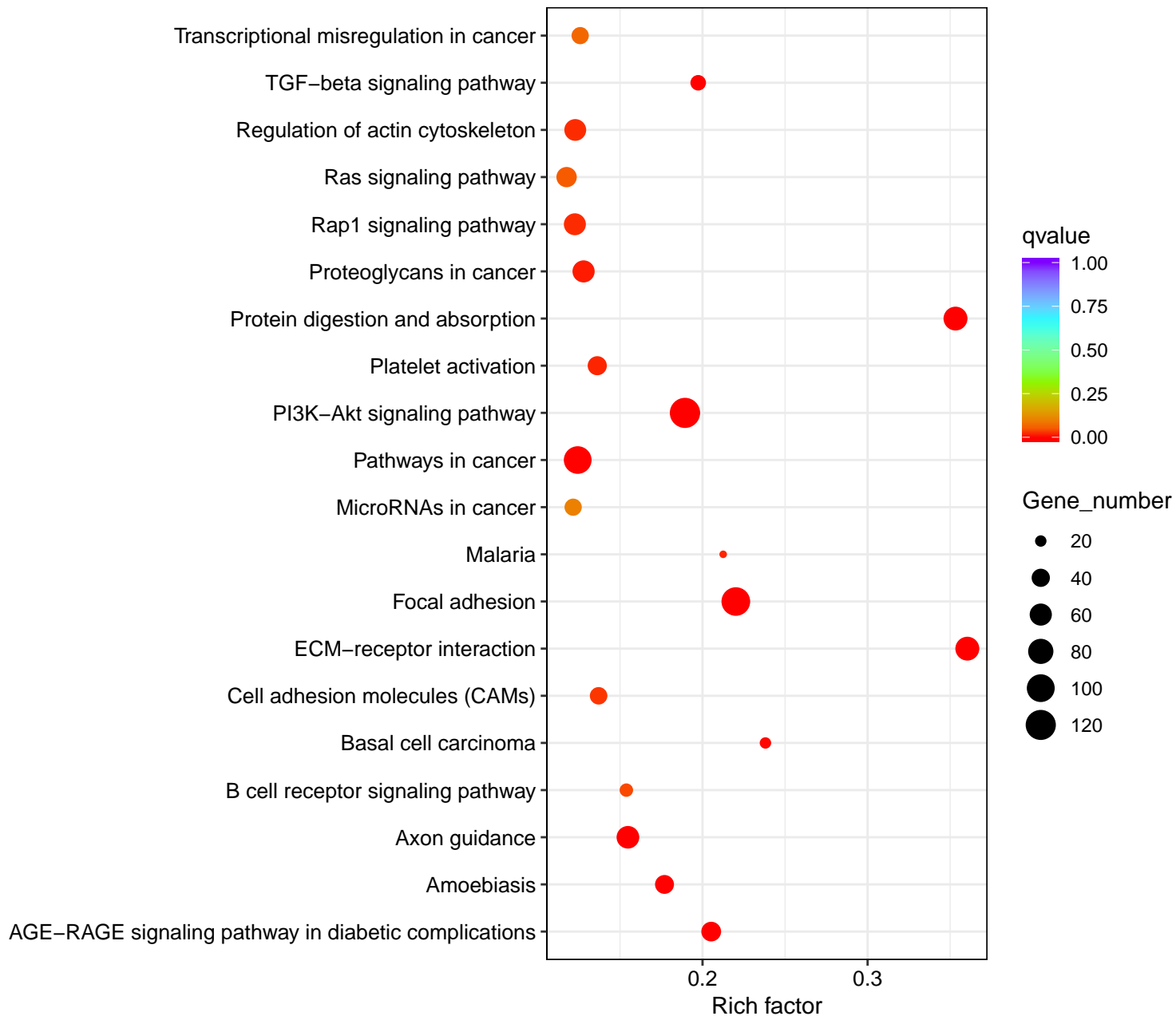

Supplement: Supplementary file 4 — Additional file 4. [file 12864_2024_10047_MOESM4_ESM.pdf]

# D.vs.C

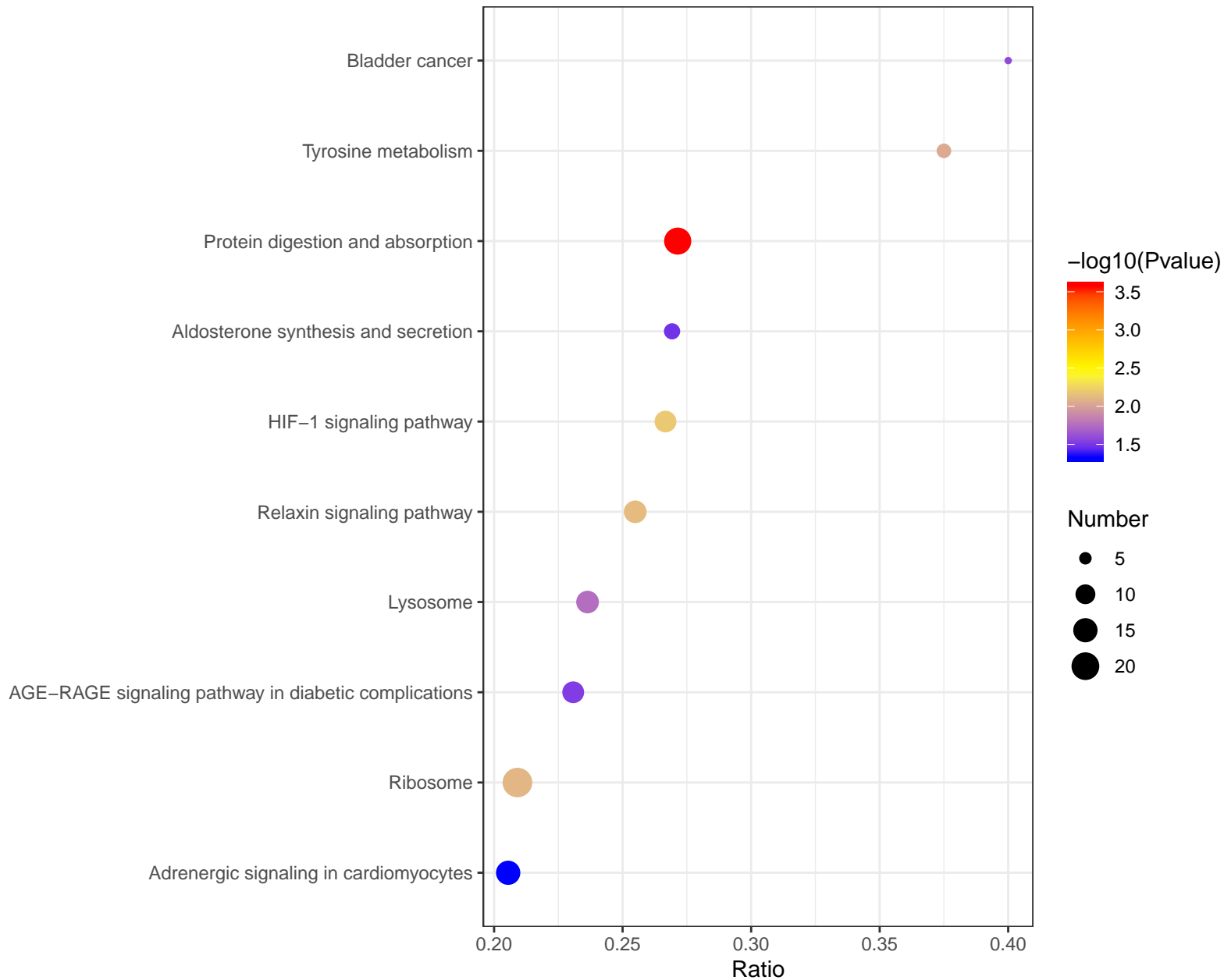

Supplement: Supplementary file 5 — Additional file 5. [file 12864_2024_10047_MOESM5_ESM.pdf]
