# Supplementary material for: Transcriptomic and proteomic strategies to reveal the mechanism of Gymnocypris przewalskii scale development
Source: BMC Genomics. 2024 Feb 3;25:140. doi: 10.1186/s12864-024-10047-1 (PMC10837935; doi:10.1186/s12864-024-10047-1)
Supplement: Supplementary file 6 — Additional file 6. [file 12864_2024_10047_MOESM6_ESM.pdf]

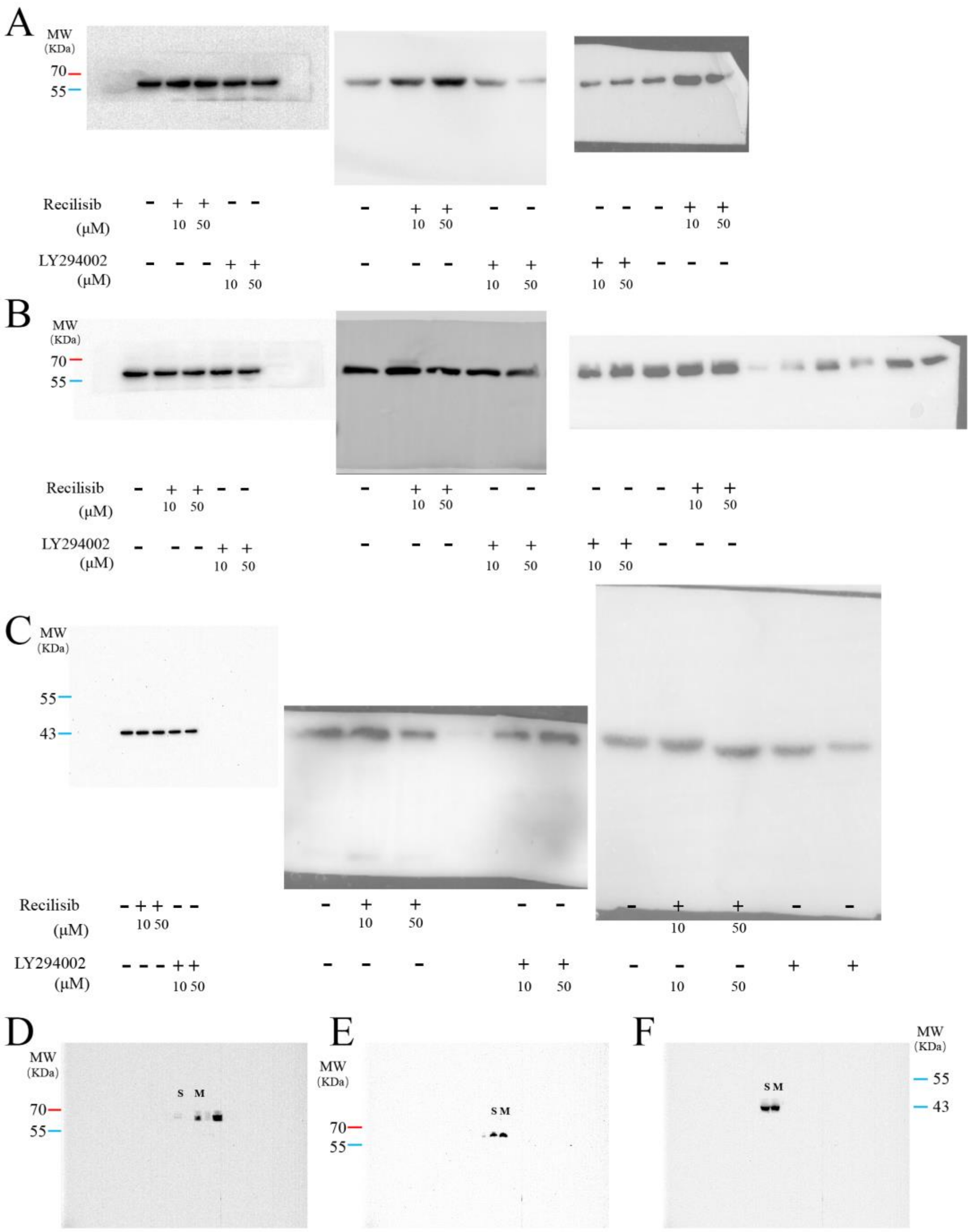

Figure S6 The original blot images corresponding to Fig. 6. (A) Blots images of all the three replicates used for protein quantification of p-Akt in Figure 6B, in which the first images were selected as a representative to illustrate in Figure 6A. (B) Blots images of all the three replicates used for protein quantification of Akt in Figure 6B, in which the first images were selected as a representative to illustrate in Figure 6A. (C) Blots images of all the three replicates used for protein quantification of β-actin in Figure 6B, in which the first images were selected as a representative to illustrate in Figure 6A. For blotting analyses of p-Akt, Akt and β-actin, the PVDF membranes were cut prior to

hybridization with specific antibodies, and placed to expected areas of the target proteins when transferred. As shown in the blot images, detections of the target proteins were highly specific as expected. (D) The un-cropped image for p-Akt Western blot analysis corresponds to Fig. 6C. (E) The un-cropped image for Akt Western blot analysis corresponds to Fig. 6C. (F) The un-cropped image for β-actin Western blot analysis corresponds to Fig. 6C.
